# Evolution of physico-chemical properties of *Dicranopteris linearis*-derived activated carbon under various physical activation atmospheres

**DOI:** 10.1038/s41598-021-93934-x

**Published:** 2021-07-13

**Authors:** Nga T. Mai, Minh N. Nguyen, Toshiki Tsubota, Phuong L. T. Nguyen, Nam H. Nguyen

**Affiliations:** 1 Faculty of Environmental and Natural Resources, Ha Tay Community College, Thuy Xuan Tien, Chuong My, Hanoi, Vietnam; 2 Faculty of Environmental Sciences, University of Science, Vietnam National University, Hanoi (VNU), 334 Nguyen Trai, Thanh Xuan, Hanoi, Vietnam; 3 Department of Materials Science, Graduate School of Engineering, Kyushu Institute of Technology, 1-1 Sensuicho, Tobata-ku, Kitakyushu, Fukuoka, 804-8550 Japan; 4 Department of Mechanial Engineering, Can Tho University, 3/2 street, Can Tho City, Vietnam; 5 Energy Department, University of Science and Technology of Hanoi, Vietnam Academy of Science and Technology, 18 Hoang Quoc Viet, Cau Giay, Hanoi, Vietnam

**Keywords:** Structural properties, Materials science, Biomaterials

## Abstract

This work emphasizes the effect of the physical activation using CO_2_ and steam agents on the physicochemical properties of activated carbon produced from *Dicranopteris linearis* (*D. linearis*), a fern species widely distributed across tropic and subtropic ecoregions. The *D. linearis*-derived chars produced under pyrolysis at 400 °C for 1 h were activated in various CO_2_-steam proportions. As revealed by the IR and Raman spectra, the structure of the activated chars was heavily dependent on the relative proportion of CO_2_ and steam. The total specific surface area (SSA) of the activated chars proportionally increased with the increase in steam proportion and was comparable to the values of commercial activated char products. Specifically, the activation under CO_2_^−^ and steam-saturated conditions has correspondingly resulted in SSA increasing from 89 to 653 m^2^g^−1^ and from 89 to 1015 m^2^g^−1^. Steam also enhanced the development of mesoporous structures of the *D. linearis*-derived char products, thereby extending their potential applications, particularly for industries that require high rigidity in the product such as pharmaceutical and cosmetic sectors.

## Introduction

Activated carbon has received much attention in recent years thanks to its high adsorption capacity^1–3^. This material has been applied in a wide range of advanced industrial applications such as waste treatment, metal production, electrodes for supercapacitors, cosmetics and pharmaceutical industries^1,4^. The quality and properties of activated carbon, that are crucial for extending their application potentials, have been demonstrated to depend on the nature of input feedstocks and activation methods. Basically, activated carbon can be produced by two common methods, namely chemical and physical activation^3^. Chemical activation is a single-step method that impregnates raw materials with activating agents, followed by heat-treatment in an inert atmosphere^5^. Various activating agents such as H_3_PO_4_, ZnCl_2_, K_2_CO_3_, KOH have been attempted to promote the elimination of impurities in the pore system, prevent mass loss, improve surface area and boost the yield of char products^6–8^. However, these chemical activation approaches will inevitably cause various environmental concerns, particularly the excessive uses of the corrosive activating agents and possible releases of the agents in the purification (washing and drying) steps^9^.

In contrast to chemical activation, physical activation is a more environmentally friendly approach that allows char production without any secondary waste disposal steps. Physical activation involves the carbonization of raw materials, followed by the activation of resulting chars with different activating agents such as air, carbon dioxide, steam, or their mixture^10^. By condensing a certain amount of internal carbon mass, a well-developed carbonaceous structure with a high surface area can be obtained. Following these structural changes, heavy metal impurities are also inadvertently removed^11^. Therefore, physical activation likely better fits for purposes that require a more stringent health aspect. Physical activation is a delicate process that is subjected to various factors such as feedstock pre-treatment, carbonization condition, nature of activating agents and activation temperature^10–13^. Among these factors, activating agent (type and concentration) can be one of the most decisive factors in determining char’s characteristics. Air can also be used as an activating agent^14^. However, it reacts more aggressively with carbon, leading to burnout inside of pore structures and resulting in lower product yield^15^.

The individual or mutual application of CO_2_ and steam for activation is potential thanks to their lower reactivities with carbon. The activation using steam was proved to be cost-efficient in producing biomass-derived activated carbon that can substitute costly commercial adsorbents^14^. Having abundance of hydroxyl (–OH) and carboxyl (–COOH) groups and high specific surface area (SSA), steam-induced activated carbon serve as a good adsorption capacity^15,16^. In comparison with CO_2_, steam presents a better performance in enhancing microporosity and mesoporosity^17^, SSA and reactive surface functional groups^18^. It is noteworthy that most of the existing studies solely focused on either CO_2_ or steam activation, while few attempts have been conducted to combine these activating agents. In reality, it is difficult to obtain an activation environment that consists of either CO_2_ or steam individually. When a biomass feedstock is decomposed under the effect of temperature, certain amounts of CO_2_ and steam will be co-generated^16^. Therefore, the mixture of steam and CO_2_ is commonly presented in a real process. In this environment, the char conversion will be mutually affected by these agents. Guizani et al.^19^ reported a synergistic effect on the conversion of the beechwood biochar under the mixed atmosphere, in which the characteristics of the final char products were also modified greatly. On the contrary, a competition between CO_2_ and steam on the reactive sites of coal chars was also reported^20^. The mutual effects of CO_2_ and steam on different raw materials are, therefore, less predictable. Hence, further empirical experiments are highly required to elucidate to what extent the structure of char can be modified at a wide range of CO_2_—steam activation atmospheres.

This study aims to evaluate the characteristics of the fern *Dicranopteris linearis* (*D. linearis*) and its activated chars produced in different CO_2_-steam activation atmospheres. The fern *D. linearis*, which is predominant in humid subtropical and tropical regions^21^, was selected to produce activated carbon. This fern species recycles annually, thereby potentially serving as an abundant source of biomass. In many remote regions, *D. linearis* is commonly burned to return ash and its accompanying nutrients to the farmland, while the remaining part of this herbaceous plant is considered as a fuel source for cooking by local people^22^. However, due to the low economic value of these traditional usages, expanding its applications plays a crucial role to take advantage of this abundant biomass, as well as helping improve the local people’s income. Moreover, the fern *D. linearis-*derived biochars expose a high fixed-carbon content (up to 25% wt.) and high porosity (up to 700 m^2^g^−1^)^22^, encouraging the idea of producing activated carbon. More importantly, the production of activated carbon from *D. linearis* could be an innovative approach to open high-value applications for this wild plant.

## Materials and methods

### Sample preparation

*Dicranopteris linearis* was collected in a hilly area at Trung Khanh district, Cao Bang province, Vietnam (Figure S1) under the permission from Vietnamese Government. The research experimental protocol in this manuscript had complied with all current guidelines and legislation of Vietnam. The plant sample was harvested from three separate plots (1 m × 1 m) and mixed together to form a composite sample. The sample was washed with deionized water, air-dried, ground, and then passed through a 1-mm sieve prior to analysis and preparation of biochar.

### Physio-chemical characteristics of D. linearis

The main physicochemical characteristics of *D. linearis* were analyzed to obtain a more in-depth understanding, serving for the preliminary assessment of the potential of this material. The structure of *D. linearis* plant (stem and petiole) was visualized by synchrotron-based X-ray tomographic microscopy (SRXTM) at the TOMCAT beamline, Swiss Light Source, Paul Scherrer Institute, Switzerland to provide a three-dimensional image of the principal arrangement of organic matters for a vascular bundle in different plant parts. Proximate analysis of *D. linearis* was carried out to determine the volatile (V) matter (ASTM D-3175), ash (A) content (ASTM D-3174), and fixed carbon (FC) content (FC% = 100 − V − A). An elemental analyzer (PerkinElmer 2400 Series II) was used to determine the elemental compositions of the plant.

**Figure 1:**
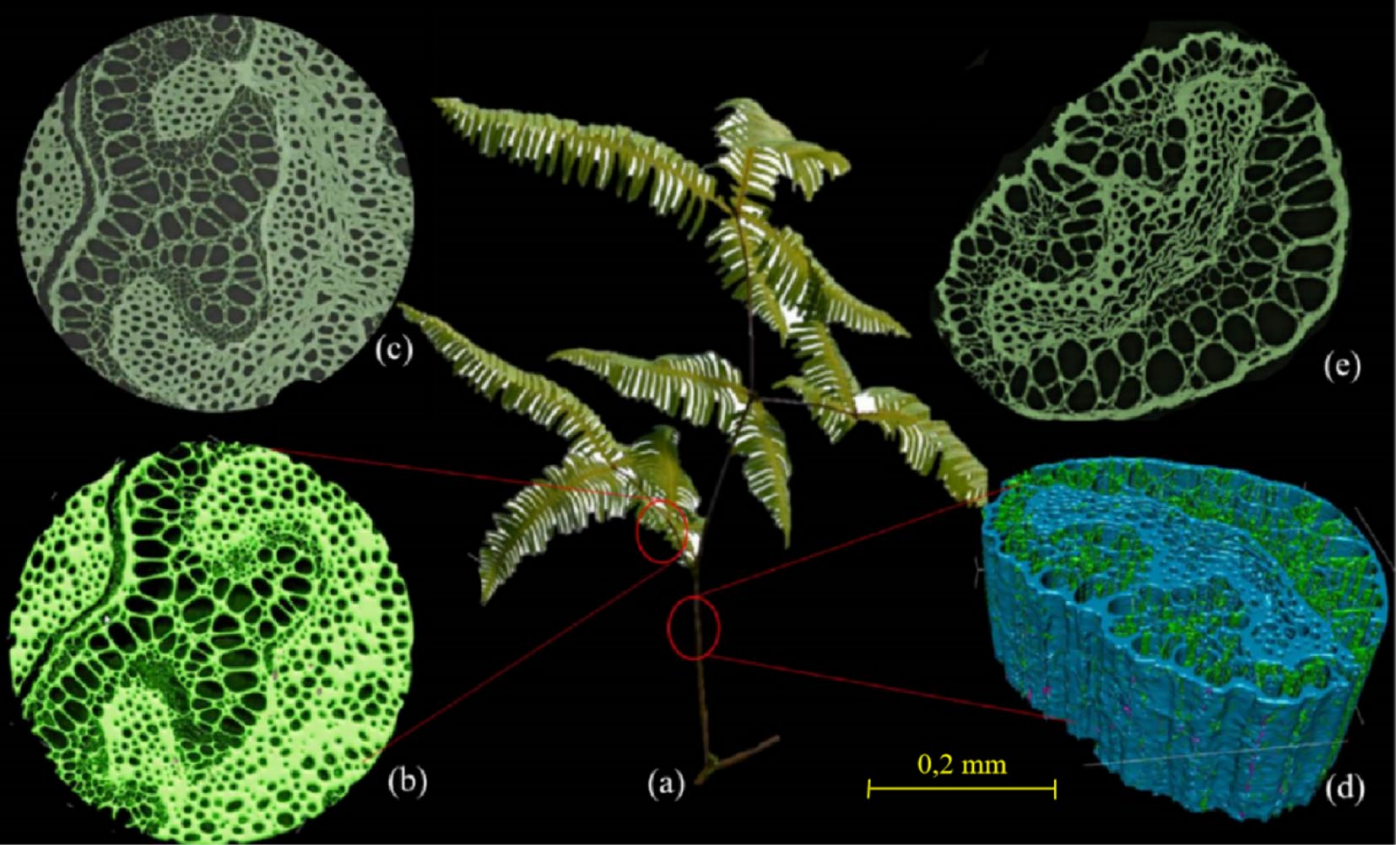
Schematic description of (**a**) *D. linearis* biomass and tomography 3D images showing different parts: (**b**, **c**) petiole and (**d**, **e**) stem.

The principal arrangement of organic matter for a vascular bundle in different parts of the plant (stem and petiole) was visualized in Fig. 1. The structural traits of the *D. linearis* stem and petiole present a highly porous pattern with various sized pores that reflect the longitudinal vascular bundles, veins, or fibres in the stem and petiole in *D. linearis.* This porous structure could be favorable for converting *D. linearis* by thermochemical methods since heat can be transferred to different parts more quickly and evenly. The proximate and ultimate results of *D. linearis* are reported in Table 1. It is noteworthy that the *D. linearis* has much higher FC content compared to other common biomass types^23^, making it a promising material for fabrication of biochar and activated carbon.

**Table 1:** Proximate and ultimate analysis of *D. linearis.*

V_db_%	A_db_%	FC_db_%	C_daf_%	H_daf_%	O_daf_%	N_daf_%
66.8	8.3	24.9	55.7	5.7	38.4	0.2

*V* volatile, *A* ash, *FC* fixed carbon, *HHV* higher heating value, *db* dry basis, *daf* dry ash-free basis.

### Char production

**Figure 2:**
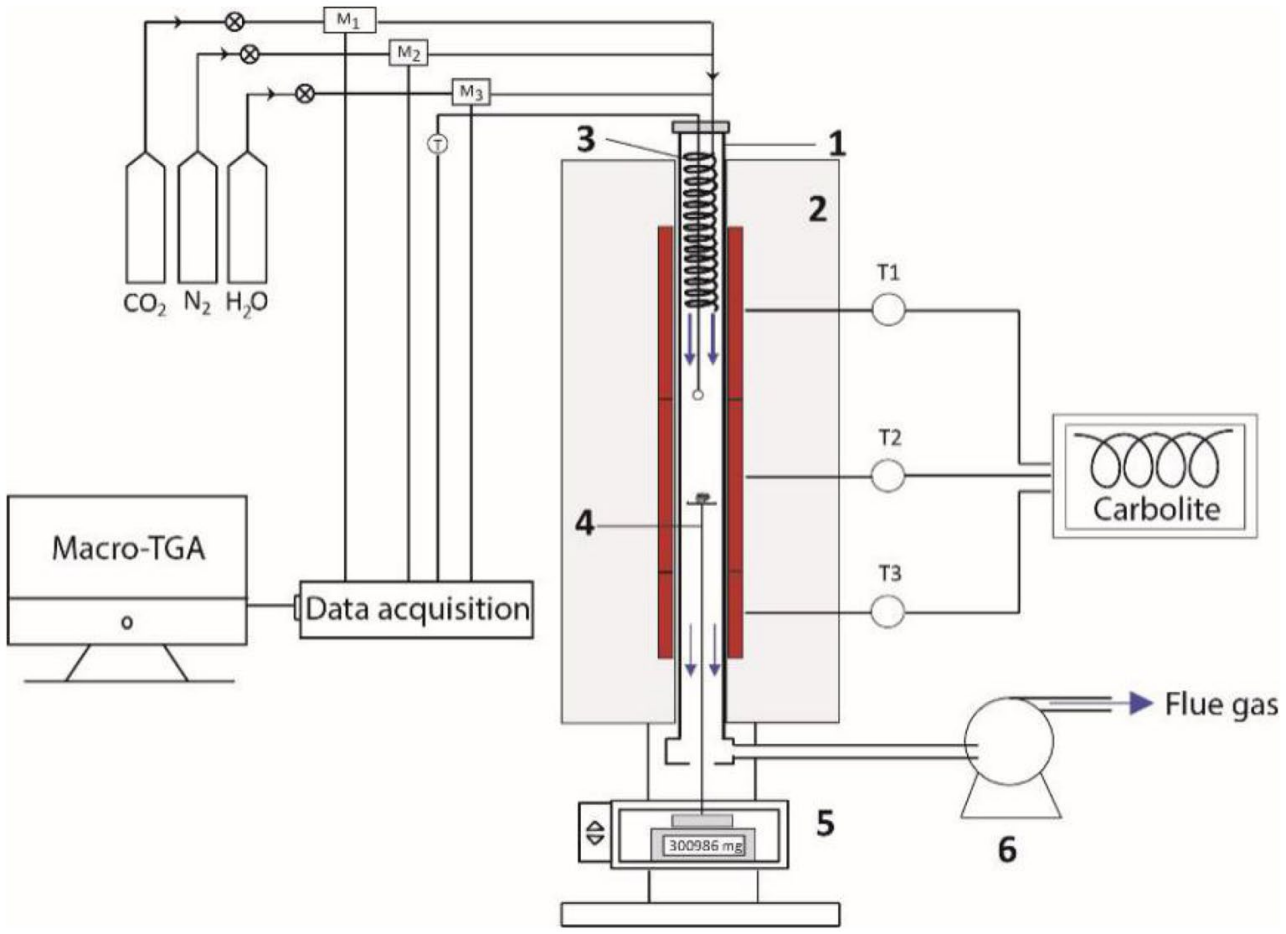
The macro-thermogravimetric reactor: (1) Ceramic tube, (2) Electrical furnace, (3) Preheater, (4) Sample holder, (5) Weighing scale, and (6) Gas extractor.

The production of char was performed in an electric muffle furnace (Nabertherm LT 24/12/P300). Approximately 300 g of *D. linearis* was put into an airtight steel box (10 cm in height and 20 cm in diameter) under a continuous flow of N_2_ (3 Nl min^−1^) (Fig. 2). The pyrolysis process was set up at a heating rate of 15 °C min^−1^ and maintained at a temperature of 400 °C for 1 h. This setup allowed us to obtain enough homogeneous amounts of char for further experiments.

### Activation process

A macro-thermogravimetric reactor^24^ was designed by the French Agricultural Research Centre for International Development (CIRAD) and the University of Science and Technology of Hanoi (USTH) (Fig. 2). The reactor containing a ceramic tube (1) with a length of 111 cm and an internal diameter of 7.5 cm was placed in an electrical furnace (2) with three independent heating zones (T1, T2, and T3) to stabilize temperature. The reaction atmospheres were generated by varying proportions of N_2_, CO_2_, and steam with the help of flow meters (M_i_) controlled by computer software. The gas mixture was moved across a 2-m-long coiled preheater (3) located in the upper-heated part of the reactor before reaching the sample.

For each experiment, the reactor was heated to 900 °C (activation temperature). Then the sample holder (4) containing 200 mg of the *D. linearis* derived-char was lifted to the desired position and maintained under a gas flow rate of 3 Nl min^−1^. The proportion of CO_2_ and steam was adjusted to vary from 100% CO_2_ to 100% steam. The mass change of the sample during the activation process was recorded by using a weighing system (5) connected with a computer. The exhaust gas was sucked out of the reactor by using a gas extractor (6). After half of the mass of carbon was removed, the gas flow was switched to 100% N_2_ until the reactor was completely cooled down to avoid possible oxidation of the obtained chars. The chars were then collected and subjected to various analyses to evaluate the structural and physicochemical properties.

### Characterization of char products

#### Micromorphology and chemical composition

The scanning electron microscopy (SEM) (Hitachi S-4800) coupled with energy-dispersive Xray spectroscopy (EDS) was performed to observe the surface morphology and surface elemental composition of the chars. The SEM–EDS technique allows a direct visualization and provides valuable information on the macro-porosity development and the state of the char surface.

#### *N*_*2*_* adsorption/desorption*

The porosity of the chars obtained from different conditions was analyzed by the N_2_ adsorption–desorption technique. The char samples were outgassed at 300 °C for 3 h in a vacuum, and then introduced to the BELSORP-mini II analyzer to conduct N_2_ adsorption–desorption at 77 K. Data was obtained over the relative pressure range from 0 < *p/p*_0_ < 0.99. Based on the adsorption–desorption results, the Brunauer–Emmett–Teller (BET) method was applied to estimate the SSA and total pore volume of the chars, while micropore surface area and volume were estimated by the *t*-plot method.

#### Carbon structure

A Raman spectrometer (NSR-5100, JASCO Corporation) was used to investigate the carbon structure in activated chars. This technique is useful for highly disordered carbonaceous materials such as biochar. The samples were scanned under a 5mW-laser power at a wavelength of 532 nm. The obtained Raman spectra were then separated into G band (corresponds to an ideal graphitic structure) and D bands (corresponds to defects or disorders). Detailed description of each band was presented in Figure S2. The intensity ratios of the G and different D bands were used to characterize the defect quantity in activated chars.

#### Surface functional groups

The functional groups present on the surface of activated chars were determined through the Fourier-transform infrared spectroscopy (FT-IR) method, using the device Elmer Perkins Spectrum Two. The powder of each sample was inserted into the diamond crystal port and compressed by a hammer with a compression force of about 40 N. The FT-IR spectra were recorded in the wavenumber range 4000–450 cm^−1^ with 1 cm^−1^ resolution.

## Results and discussion

### Surface morphology

**Figure 3:**
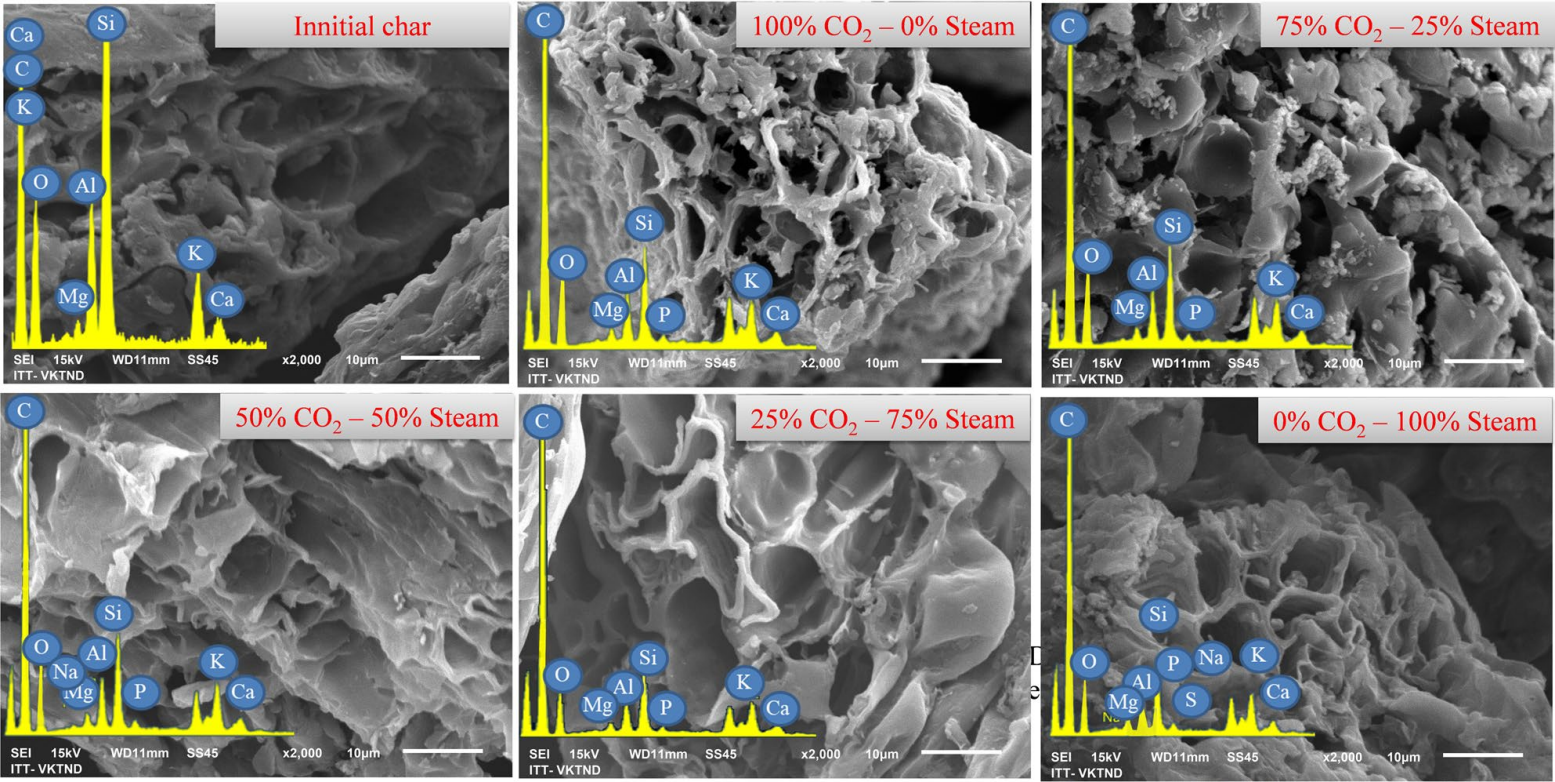
SEM images and EDX spectra of the original and activated chars.

The surface morphology and chemical composition of the original char and activated chars prepared at different proportions of the activating agents were investigated by the SEM–EDS technique (Fig. 3). The SEM image of the original char showed a less-defective surface with limited pores in comparison with the activated chars. The chemical composition measured by EDS spectra reflected the predominance of C (60–68%) and Si (3.5–5.9%) along with other minor elements such as Ca, Mg, Al, and Cu. Meanwhile, an intensification of pores in different sizes and shapes was observed for the activated chars. This highlights the development of char porosity during the activation process. Nevertheless, microstructures of the char could not be fully detectable by the SEM. Therefore, the specific effects of the activating agents on the microstructures were beyond the resolution of this method. In comparison with the original char, the activated chars showed a significant increase in C content (78–81%), and a decrease in Si content (1–3%), along with the appearance of other elements such as P and Na. These notable changes of C and Si might be explained by the reduction of Si resulted from dissolution and removal of Si from the chars under the influence of steam. Moreover, other compounds such as Al, K, Na, Mg and Fe, were still conserved under the high thermal conversion process. It is noteworthy that there was no obvious change in the surface elements for the activated chars obtained from different CO_2_-steam proportions.

### Microstructure and specific surface area

**Figure 4:**
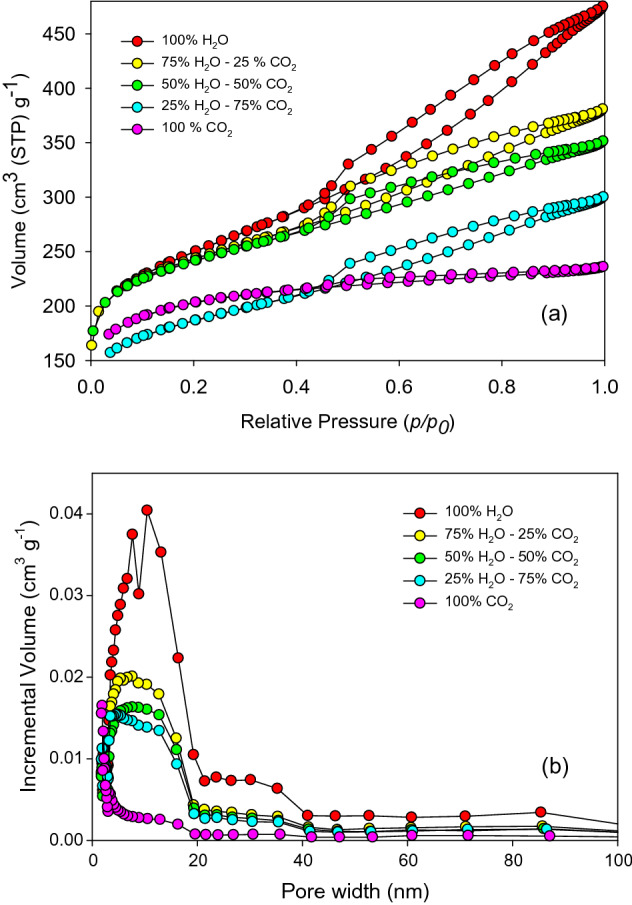
(**a**) Adsorption/desorption isotherms and (**b**) pore-size distribution of *D. linearis* chars.

The type of porous structure is ascertained based on the N_2_ adsorption/desorption isotherms for different chars (Fig. 4). For the original char, a low amount of N_2_ volume was absorbed in the pores (Fig. 4a), suggesting that the original char is less porous, or it might contains ultra-micropores (that N_2_ molecules cannot enter). For the activated chars, the adsorption results indicated the extension of the porous structure due to the reaction of CO_2_/steam and char, as proved by the increased slope at relative pressures *p*/*p*_0_ above 0.1.

The pore size distribution (Fig. 4b) showed peaks in the range below 15 nm, indicating that micropores and mesopores were dominant in the structures of the activated chars. Moreover, for the activated char produced under 100% CO_2_ condition, the isotherms are closer to type I in the IUPAC classification^25^, which is likely characteristic of microporous structures. With the presence of steam, the isotherms evolved from type I to type IV with a hysteresis loop (H4 type) in the *p*/*p*_0_ region between 0.45 and 1.0. This phenomenon is usually associated with the capillary condensation, indicating micro-mesoporous structures constituted of narrow slit pores^20^. This suggests that in addition to the micropores, the mesopores were also developed in the steam-activated chars. The results of total surface area (*S*_total_) and total pore volume (*V*_total_), estimated by BET method, micropore volume (*V*_micro_) and micropore area (*S*_micro_), estimated by t-plot method, and mesopore area (*S*_meso_) and mesopore volume (*V*_meso_), estimated by BJH method, are summarized in Table 2.

**Table 2:** Pore volumes and surface areas of activated chars.

Sample (CO_2_-steam proportion)	*S*_total_ (m^2^ g^−1^)	*S*_micro_ (m^2^ g^−1^)	*S*_meso_ (m^2^ g^−1^)	*V*_total_ (cm^3^ g^−1^)	*V*_micro_ (cm^3^ g^−1^)	*V*_meso_ (cm^3^ g^−1^)
Original char	89	39	34	0.028	0.012	0.011
100–0	653	556	77	0.473	0.160	0.159
75–25	805	571	214	0.613	0.195	0.328
50–50	957	643	270	0.651	0.135	0.317
75–25	1005	639	307	0.701	0.165	0.570
0–100	1015	663	330	0.761	0.190	0.381

The *S*_total_ showed a great leap when increasing from approximately 89 m^2^ g^−1^ for the original char to the highest value of 1015 m^2^ g^−1^ for the char activated in 100% steam. Comparing *V*_total_ and *V*_micro,_ the same trends as *S*_total_ and *S*_micro_ were observed. *V*_total_ steadily increased from 0.012 to 0.19 cm^3^ g^-1^ in the case of 100% CO_2_ activation, and to 0.381 cm^3^ g^−1^ in the case of steam activation. It is noteworthy that the *S*_total_ and *V*_total_ of the char products were increased along with the increasing proportion of steam, indicating that steam created a more porous char thanks to higher mesoporous structures. This result can be explained by the fact that water molecules of steam possess smaller size compared to CO_2_ molecules, and they can diffuse more easily in the carbon matrix, catalyze the reaction more effectively, thereby predominantly creating mesopores.

The SSA and pore volume of the activated chars are comparable with those of commercial activated carbons^26,27^, showing great interest in producing activated chars from *D. linearis*. Compared with various *D. linearis-*derived chars from different pyrolysis techniques in our previous studies, which showed their SSA values in the range from 40 to 524 m^2^ g^−1^, the CO_2_/steam-activated chars expressed a much higher SSA^22^. More interestingly, the performance of CO_2_ and steam in boosting SSA was at least equal to that of H_3_PO_4_^28^, and this finding is well aligned with previous studies using the same method^29,30^.

### Carbon structure

**Figure 5:**
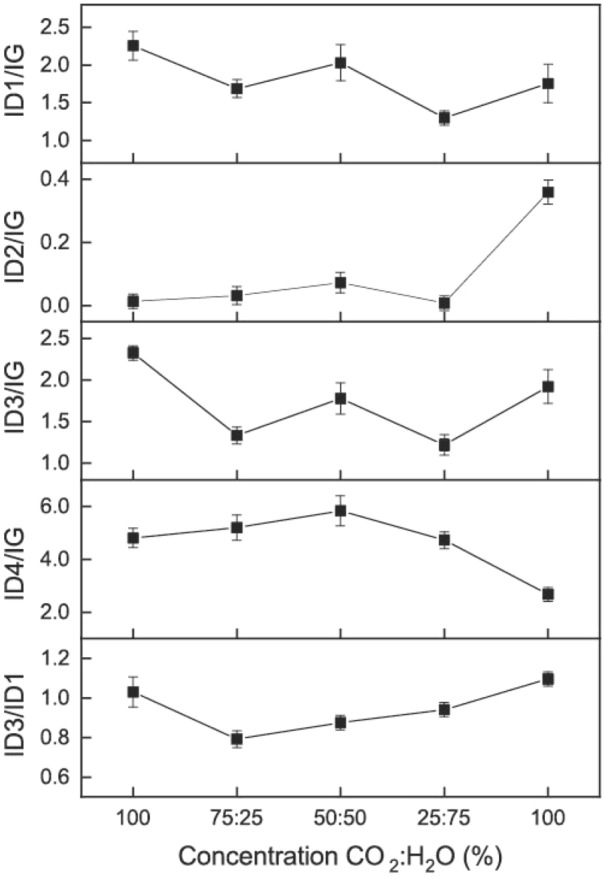
Ratios between major band intensities of activated chars.

The Raman spectra of the chars produced at different CO_2_-steam proportions were represented by the five Gaussian bands. The ratios between major band intensities were used to investigate possible structural evolution of the chars (Fig. 5). For *D. linearis*, the intensity ratio D1/G tended to decrease with the increase in steam proportion, possibly denoting the preferential reaction of steam with D1-type. The intensity ratio D2/G was stable in most of the case and then increased significantly for the case of 100% steam, resulting in an increase in the proportion of graphene layers which are not sandwiched between two other ones. The intensity ratio D3/G along with the changes in CO_2_-steam proportion was unclear. This might be due to the overwhelming reaction of steam with the poorly organized materials (D3-type). The D4 band represents *sp*^2^-*sp*^3^ sites at the periphery of crystallites and/or C–C, C=C polyne-like structures. The intensity ratio D4/G showed an increasing trend when the steam proportion < 50% and then decreased considerably after that, possibly resulting from the increase in the reactivity of these structures under the higher concentration of steam. The intensity ratio D3/D1 represents the ratio of small and large aromatic rings. This ratio has generally increased along with the increasing steam proportion. This phenomenon can be explained by the fact that when the CO_2_ concentration is high, the smaller aromatic rings were converted into larger ones or consumed. While with the high steam concentration, the bigger aromatic rings turned into smaller ones again^31^. Altogether, these results showed that the structure of the activated chars was heavily dependent on the concentration of the activating agents. Steam was predominant over CO_2_ in the mixed atmosphere, leading to the similarity in the trend of the carbon matrix when steam was present.

### Surface chemical bonds

**Figure 6:**
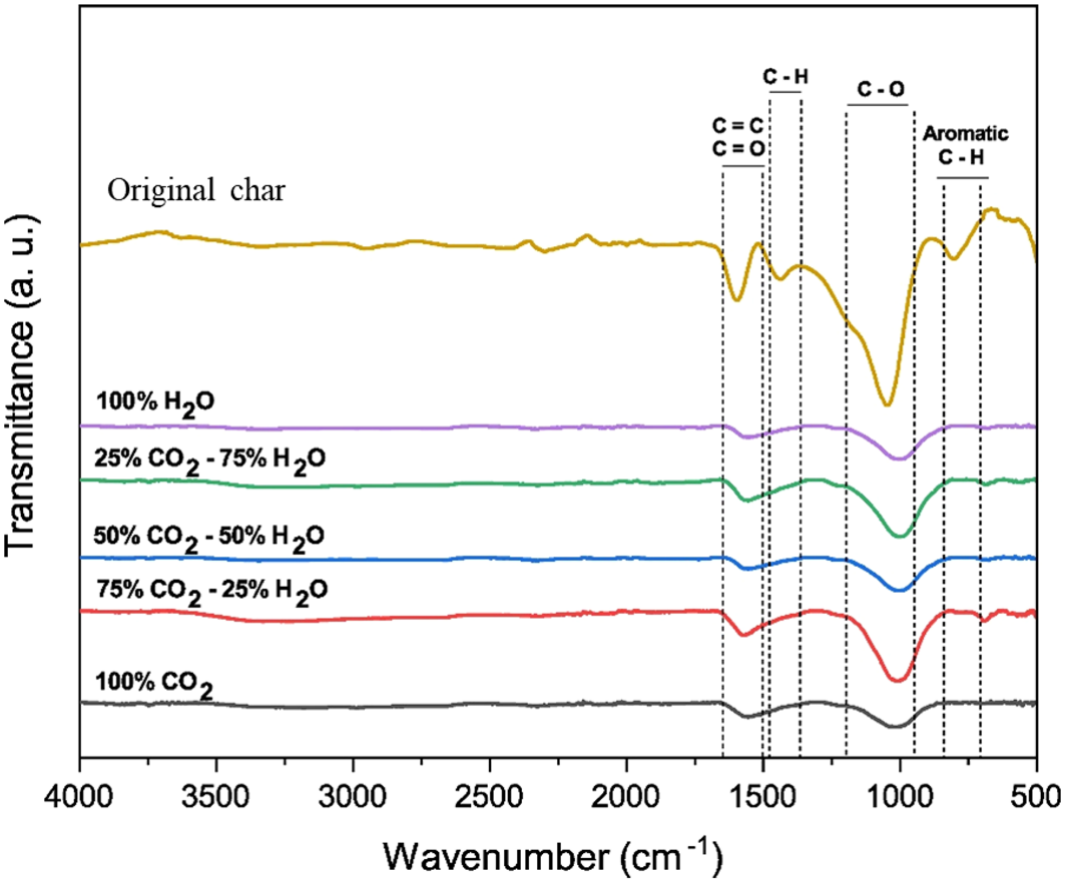
FT-IR spectra of activated char from *D. linearis* derived chars.

The IR spectra reflect the alteration of the activated chars produced under different activation conditions (Fig. 6). In general, the functional groups of the original char decreased when subjected to the activation process. The peaks at 1050 cm^−1^ could be attributed to the carbonyl groups characterized by C–O vibration, while the peaks in the range of 1600–1700 cm^−1^ could be attributed to the stretching vibrations of the functional groups C=O and/or C=C. The peak position at around 1500–1600 cm^−1^ were almost the same, suggesting that CO_2_ and steam did not alter the C=O and C=C bonds. The peaks at 800 cm^−1^ and 1450 cm^−1^ could be attributed to the hydrocarbons characterized by C–H vibrations. The intensity of these peaks was found to be strong for the original char, however, it became negligible in the case of the activated chars. This can be explained by the removal of the remained volatile compounds and some of the carbon contents in the original char under the influence of CO_2_ and steam. Moreover, the C=C bond could be dissociated when the chars were suffered from high temperatures with the sufficiency of energy. This favored the formation of unsaturated bonds among carbon atoms in the activated chars, leading to low intensity peaks (of C=C bonds). Having limited surface functional groups, the *D. linearis*-derived activated carbons seem not suitable for applications that require highly-reactive surfaces (e.g., water treatment, soil amendment). However, for applications requiring “clean” materials, e.g., cosmetic or pharmaceutical formulations, the *D. linearis*-derived activated carbons could be a potential candidate.

## Conclusions

This work highlights the potential of producing high-quality activated carbons from *D. linearis* using the physical activation method. The CO_2_-steam mixed atmospheres coupled with high temperatures was demonstrated as a suitable environment to promote the formation of highly porous chars. The SSA and pore volume the *D. linearis*-derived char products increased proportionally with the increase in steam concentration. While microporous structures were developed during activation with both CO_2_ and steam, mesoporous structures were mainly developed under the steam environment. The findings are helpful in “designing” the properties of the activated carbon by simply adjusting the proportion of steam and CO_2_. Considering the complexity of the physical activation process, the influence of other factors such as heating rate, activation temperature, activation time or their mutual effects on the properties of activated carbon need to be further investigated in future studies. Additionally, pilot tests prior to large-scale production to evaluate the effects of heat and mass transfers are also recommended.

## Supplementary Information

Supplementary Information 1.

Supplementary Information 2.
