# Supplementary material for: Evolution of physico-chemical properties of Dicranopteris linearis-derived activated carbon under various physical activation atmospheres
Source: Sci Rep. 2021 Jul 13;11:14430. doi: 10.1038/s41598-021-93934-x (PMC8277782; doi:10.1038/s41598-021-93934-x)
Supplement: Supplementary file 1 — Supplementary Information 1. [file 41598_2021_93934_MOESM1_ESM.docx]

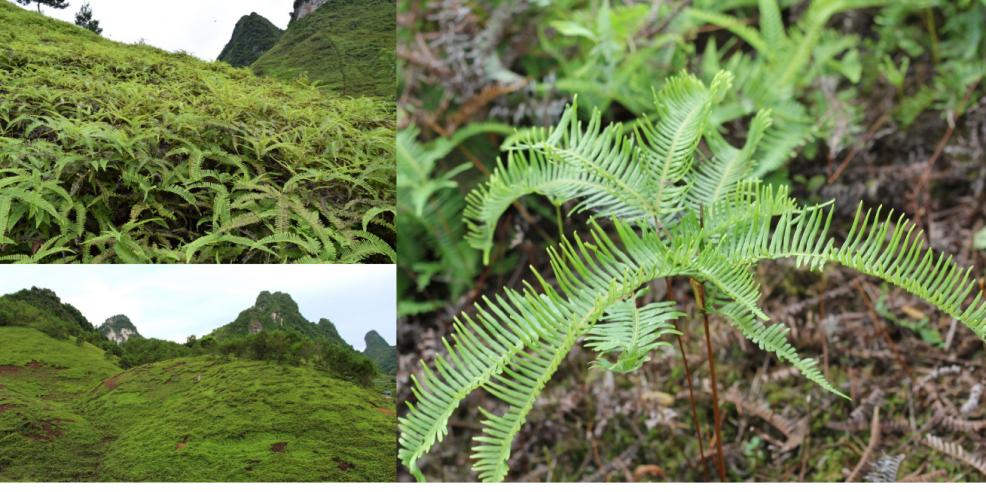


Figure S1. Sampling site at a hill densely covered with *D. linearis* in Trung Khanh district, Cao Bang province, Vietnam
