# Supplementary material for: Evolution of physico-chemical properties of Dicranopteris linearis-derived activated carbon under various physical activation atmospheres
Source: Sci Rep. 2021 Jul 13;11:14430. doi: 10.1038/s41598-021-93934-x (PMC8277782; doi:10.1038/s41598-021-93934-x)
Supplement: Supplementary file 2 — Supplementary Information 2. [file 41598_2021_93934_MOESM2_ESM.docx]

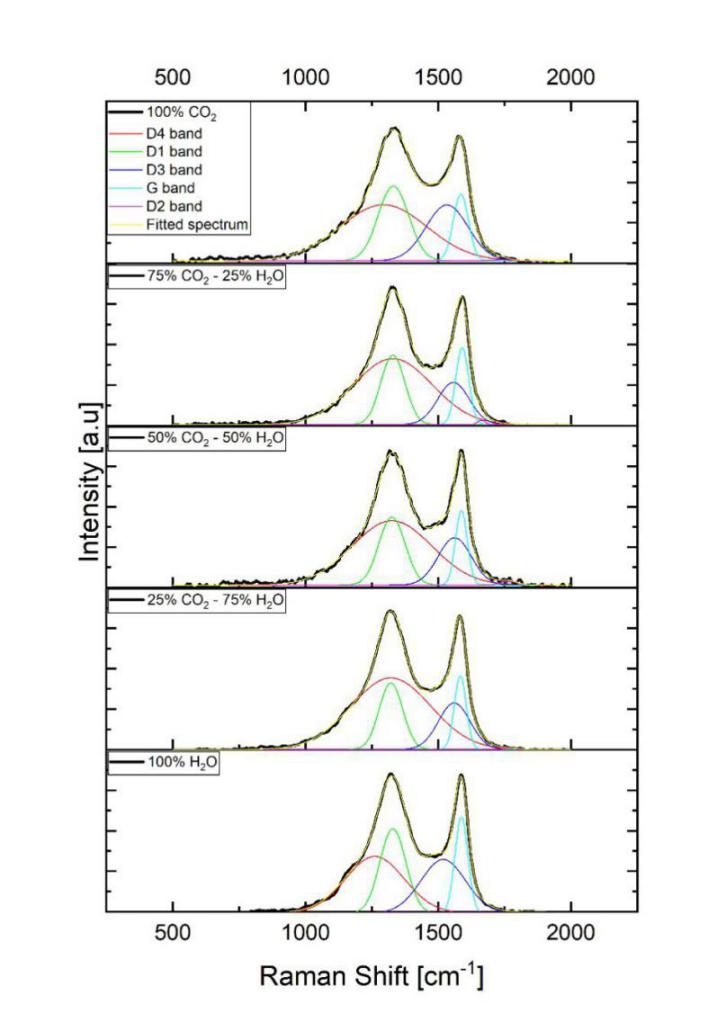


Figure S2. Raman shift of the activated chars

The G band (around 1,590 cm^-1^) corresponds to the mode *E2g* in the aromatic layers of the graphite crystalline. This is the only existing band for perfect graphite. The D1 band (around 1,350 cm^-1^) corresponds to graphitic lattice vibration mode with *A1g* symmetry. The D2 band (around 1,620 cm^-1^) corresponds to the stretching vibration mode with *E2g* symmetry in the graphene layers. The D3 band (around 1,500 cm^-1^) corresponds to amorphous carbons (organic molecules, fragments, functional groups). It is related to reactive sites and usually found in poorly organized materials. The D4 band (around 1,200 cm^-1^) corresponds to hydrocarbons. It is related to reactive sites and usually found in very poorly organized materials.
